# Bone marrow mesenchymal stem cells reduce ureteral stricture formation in a rat model via the paracrine effect of extracellular vesicles

**DOI:** 10.1111/jcmm.13744

**Published:** 2018-07-11

**Authors:** Jintai Luo, Shankun Zhao, Jiamin Wang, Lianmin Luo, Ermao Li, Zhiguo Zhu, Yangzhou Liu, Ran Kang, Zhigang Zhao

**Affiliations:** ^1^ Department of Urology & Andrology Minimally Invasive Surgery Center Guangdong Provincial Key Laboratory of Urology The First Affiliated Hospital of Guangzhou Medical University Guangzhou China; ^2^ Department of Urology The First Affiliated Hospital of University of South China Hengyang China

**Keywords:** extracellular vesicles, fibrosis, mesenchymal stem cell, ureteral stricture

## Abstract

With no effective therapy to prevent or treat ureteral stricture (US), a multifactorial fibrotic disease after iatrogenic injury of the ureter, the need for new therapies is urgent. Mesenchymal stem cells (MSCs) have been widely studied for treating tissue defects and excessive fibrosis, and recent studies established that one of the main therapeutic vectors of MSCs is comprised in their secretome and represented by extracellular vesicles (EVs). Thus, we have determined to explore the specific role of MSCs‐derived EVs (MSC‐EVs) treatment in a pre‐clinical model of US. The results firstly showed that either a bolus dose of MSCs or a bolus dose of MSC‐EVs (administration via renal‐arterial) significantly ameliorated ureteral fibrosis and recuperated ureter morphological development in a US rat model. We confirmed our observations through MSCs or MSC‐EVs treatment alleviated hydronephrosis, less renal dysfunction and blunted transforming growth factor‐β1 induced fibration. Due to MSC‐EVs are the equivalent dose of MSCs, and similar curative effects of transplantation of MSCs and MSC‐EVs were observed, we speculated the curative effect of MSCs in treating US might on account of the release of EVs through paracrine mechanisms. Our study demonstrated an innovative strategy to counteract ureteral stricture formation in a rat model of US.

## 1 INTRODUCTION

Ureteral stricture (US), defined as a narrowing of the ureter with urine flow obstruction, is a major concern in urology as renal function may be silently affected.^1^ Iatrogenic strictures after gynaecologic/urologic operations are the most common etiologies contributing to US, accounting for 35% incidence rates of all strictures.^2^ At present, various surgical options such as ureteral dilatations, endoureterotomy, ureteral stents, end‐to‐end uretero‐ureteral anastomosis, ureteroneocystostomy, ileal interposition graft and autotransplantation are employed to manage US. Endoscopic techniques, such as ureteral dilatations and endoureterotomy, represent the most common and less invasive surgical procedures for treatment of US. Although short‐term effectiveness, however, they are characterized by 40%‐60% failure rates on long‐term follow up.^3^, ^4^ Uretero‐ureteral anastomosis and ureteroneocystostomy represent valid and successful treatments for complicated US. However, the major drawback of these techniques is the recurrent US because of the formation of hyperplastic muscle or scar tissue in anastomotic stoma.^2^ Anti‐fibrotic drugs, such as glucocorticoids,^5^ captopril^6^ and halofuginone^7^ have been tested to limit re‐stricturing after US surgery. Regrettably, none of these drugs exhibit validation of therapeutic benefit thus have not been applied in the clinic.

Mesenchymal stem cells (MSCs) have been widely studied for treating tissue defects and excessive fibrosis, due to their ability to differentiate into cells needed for tissue repair and secrete a broad range of bioactive molecules (eg, growth factors, cytokines and chemokines).^8^, ^9^ However, with the recognition that only limited MSCs are recruited in the injury site after MSCs transplanted,^10^ many investigators have suggested its therapeutic effects on tissue repair might be rather by stimulating the activity of tissue‐resident recipient cells via paracrine mechanisms.^11^, ^12^

Extracellular vesicles (EVs) are small membrane vesicles originating from multivesicular bodies, which have been identified as a new kind of major paracrine factor naturally secreted by cells.^12^, ^13^ EVs can be categorized into exosomes (40‐200 nm), microvesicles (50‐1000 nm) and apoptotic bodies (50‐5000 nm), etc.^14^

Recently, the therapeutic capacity of MSCs‐derived EVs (MSC‐EVs) to treat fibrosis has been studied extensively. These cell‐free agents have shown promise in decreasing fibrosis in pre‐clinical models of lung fibrosis, liver fibrosis, cardiac fibrosis, kidney fibrosis and skin fibrosis.^15^, ^16^, ^17^, ^18^, ^19^

Based on above information, we hypothesized that MSC‐EVs such as bone marrow MSCs‐derived EVs could reduce US formation. With this scope, we try to develop a rodent model mimicking US after iatrogenic ureteral injury and to investigate the efficacy of administration of MSC‐EVs to prevent fibrosis.

## 2 MATERIALS AND METHODS

### 2.1 MSCs and MSC‐EVs preparation

Sprague Dawley (SD) rat bone marrow MSCs line (Cat. No SCSP‐402) was obtained from the Cell Bank of Chinese Academy of Sciences (Shanghai, China). MSCs were cultured in α‐Minimal Essential Medium (Gibco/Thermo Fisher Scientific) with 1% L‐glutamine supplemented with 10% Exosome‐depleted foetal bovine serum (System Biosciences, NO. 082615). Once at 80% confluence, MSCs were starved (replaced with serum‐free medium) overnight and then the conditioned medium was collected. Then, MSCs‐EVs from the supernatant were isolated by the ExoQuick TM (System Biosciences) reagent. EVs were further detected by a transmission electron microscopy analysis (Hitachi, Japan), nanoparticle tracking analysis (Malvern NanoSight, UK) and EVs characteristic surface marker proteins (CD9, CD63 and CD81). For subsequent animal experiment, a dose of 3.5 × 10^6^ MSCs from a 100 mm cell culture dish (80% of cell confluent) was administered for one animal; and an EVs dosing (25 μg protein, quantitated by the Micro Bicinchoninic Acid Protein Assay Kit (Pierce), diluted with 100 μL PBS) was determined by corresponding to the amount produced by 3.5 × 10^6^ MSCs equivalents from cell culture supernatants.

### 2.2 Animals and ethical approval

A total of 32 female Sprague Dawley rats (12 weeks old, 300‐330 g) were obtained from the Experimental Animal Center of Guangdong Province (Guangzhou, China). The Care and Use of Animal Research Committee of the First Affiliated Hospital of Guangzhou Medical University (Guangzhou, Guangdong, China) approved all experimental protocols on animals (Registration numbers: 2017‐021).

### 2.3 Study design

**Figure 1:**
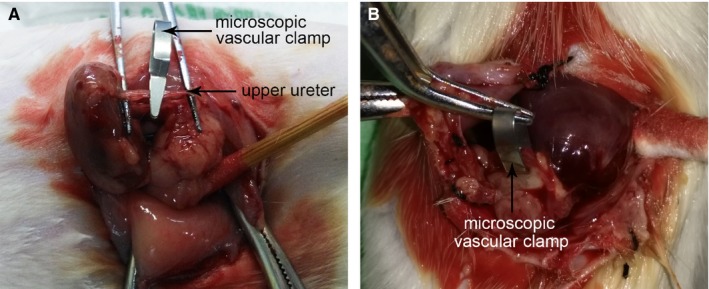
Ureteral stricture rat model. A, The upper ureter was exposed and positioned with a microscopic vascular clamp. B, After 3 d, the clamp was removed from the ureter via the initial incision

We established an original US animal model using microscopic vascular clamp placed on the left proximal ureter and subsequently removed 6 hours later. Rats were randomly divided into four groups. Sham rats had their left ureters exposed but not clamped via a flank incision. The remaining animals were subjected to establish US models. Under anaesthesia, the left ureter was visualized through a flank incision and clamped with sterile microscopic vascular clamp (15 mm; Mingmou Medical Apparatus Instruments, Suqian, Jiangsu, China) at upper ureter just below the lower pole of the left kidney (Figure 1A). For characterizing the pathogenesis of US causing by ureter damage, all the clamps were removed via the initial incision at 6 hours after surgery (Figure 1B). After clamps were withdrawn (not for Sham group), all rats received an injection in the left renal artery with either 100 μL PBS (Sham group, n = 8; US group, n = 8), 3.5 million MSCs in 100 μL PBS (MSCs group, n = 8), or 25 μg MSC‐EVs in 100 μL PBS (MSC‐EVs group, n = 8). We preferred to do the injection using a 29‐G insulin syringe (Becton, Dickinson and Company, USA) filled with 100 μL PBS/MSCs/MSC‐EVs.

### 2.4 Magnetic resonance imaging assessment

All rats were scanned by Magnetic resonance imaging (MRI) at 2 weeks and 4 weeks following the treatment for assessing the presence of hydronephrosis. Based on the MRI images (sagittal, coronal and transverse section), the left renal pelvis volume (RPV) of each rat was quantified in accordance with a previously described method,^20^ following the calculation formula: “maximum anteroposterior diameter” × “maximum length diameter” × “maximum transverse diameter” × 0.523.

### 2.5 Renal function test

Blood samples were collected from the inferior vena cava for detection of serum creatinine (Cr) and blood urea nitrogen (BUN) at 4 weeks after treatment. Cr and BUN were measured using Automated Chemistry Analyzers (Beckman Coulter Chemistry Analyzer AU5800, CA, USA) according to the manufacturer's instructions.

### 2.6 Histology

After rats were killed, the stenotic tissue of the ureter segment (proximal and distal to the injury site, about 1.5 cm) and the homolateral kidney were harvested, fixed and further processed for histology. Haematoxylin and eosin (HE) and immunohistochemical staining procedures were performed according to a standard protocol. The degree of US was evaluated by the quantification of the ureteral lumen diameter. We assessed the degree of tissue damage in the renal cortex and outer medulla (ie, tubular necrosis and tubular enlargements.) on a scale of 0 to 2 (none to marked) according to Toyohara et al's study.^21^ The scoring of immunoreactivity was obtained by adding the score of positive cell percentages and staining intensities.

### 2.7 qRT‐PCR and Western blot

The mRNA and protein expression levels of collagen I (Col I), collagen III (Col III), fibronectin (Fib), transforming growth factor‐β1 (TGF‐β1) and Smad3 (phosphorylation of Smad3 (p‐Smad3) was used in Western blot (WB)) in experimental ureter tissue samples were examined. β‐actin was employed as the internal normalization. The sequences of the PCR primers are as follows are listed in Table S1. All the relevant antibodies were purchased from Abcam Biotechnology.

### 2.8 Statistical analysis

The results were analysed using SSPS 16.0 software (SPSS, Inc., Chicago, IL) and expressed as mean ± standard deviation of the mean. One‐way analysis of variance followed by Bonferroni's multiple comparison tests was used to evaluate whether differences between groups were significant. Statistical significance was set at *P *<* *.05.

## 3 RESULTS

### 3.1 Characterization of MSC‐EVs

**Figure 2:**
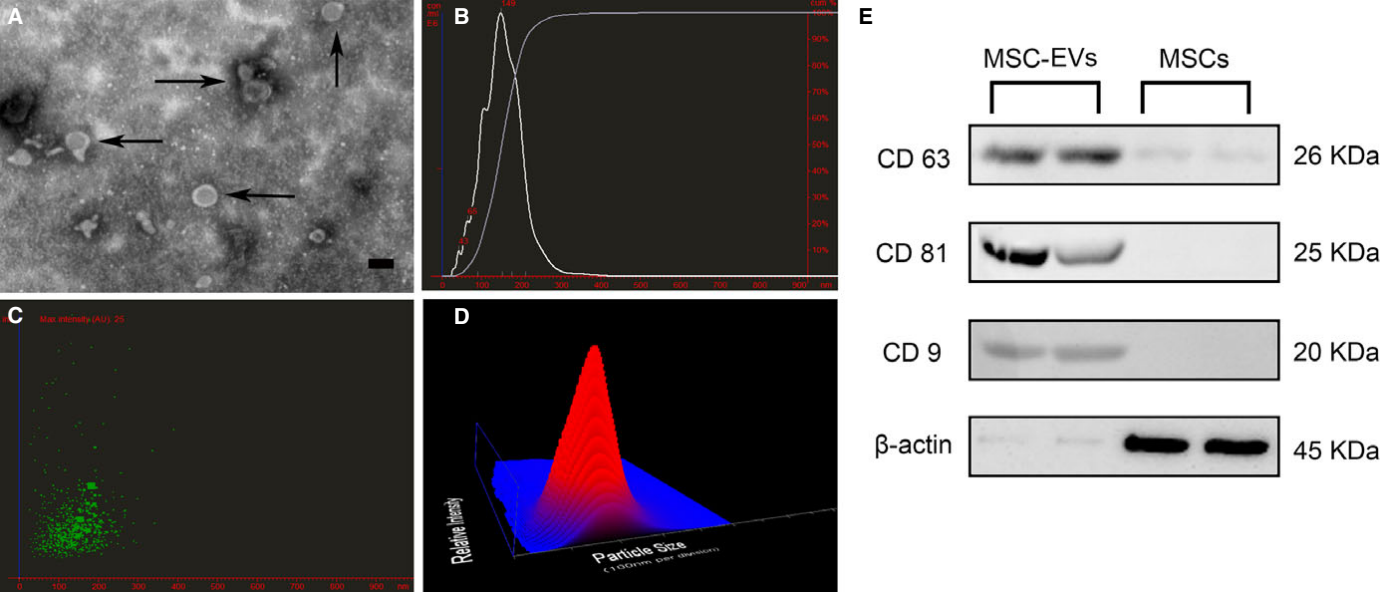
Characterization of mesenchymal stem cells derived Extracellular vesicles (MSC‐EVs). A, Transmission electron micrograph image of MSC‐EVs, Scale bar = 100 nm. The morphology of EVs was detected as grape‐like clusters of vesicles with double layer membrane structure and diameters about 120 nm. B‐D, nanoparticle tracking analysis revealed the concentration of the particles was 2.75 × 108 particles/mL; the mean diameter of the particles was 149 nm. E, Western blot results showed that EVs markers CD63, CD81 and CD9 could be detected in the protein levels

Transmission electron microscopy revealed that EVs were grape‐like clusters of vesicles with double layer membrane structure and diameters about 120 nm (Figure 2A). Nanoparticle tracking analysis demonstrated that the concentration of the particles was 2.75 × 10^8^ particles/mL; the diameters of the particles were within the range of 100‐170 nm, with a mean size of 149 nm (Figure 2B‐D). Western blot analysis of MSC‐EVs revealed the presence of EVs membrane proteins CD9, CD63 and CD81 (Figure 2E).

### 3.2 Animals

One rat of the MSCs group died due to perioperative infection after microscopic vascular clamp implantation. The body weight of rats at initial and 2 weeks after treatment did not differ significantly among the 4 groups (*P *>* *.05, for all). At 4 weeks after treatment, the body weight of the Sham group was significantly greater than that of the other three groups (*P *<* *.05, for all). The body weight of MSCs and MSC‐EVs groups increased a slight but not statistically as compared with the US group (*P *=* *1.000 and 1.000, respectively; Table S2).

### 3.3 Mri

**Figure 3:**
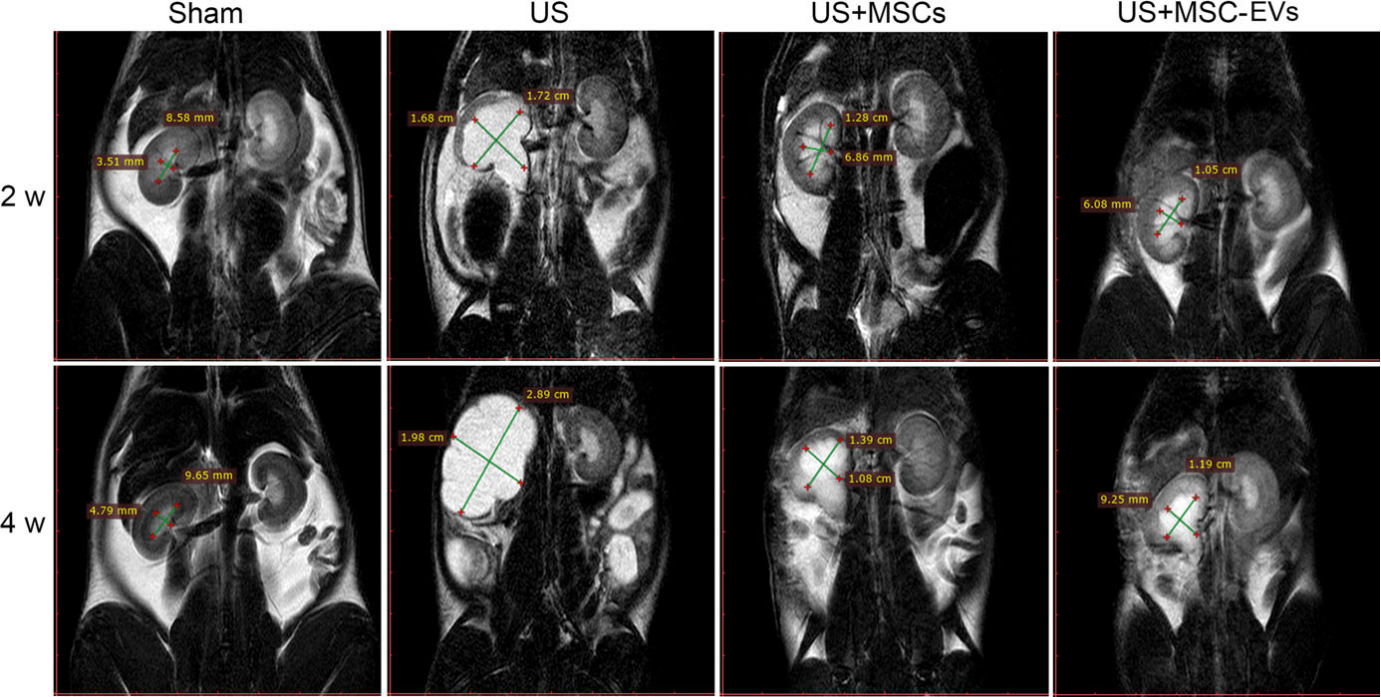
Magnetic resonance imaging (MRI) examination. Representative MRI images of hydronephrosis of a sham rat and rats from the ureteral stricture (US) group treated with vehicle, bone marrow mesenchymal stem cells (MSCs) or MSCs‐derived EVs. Upper panels and lower panels are representative MRI images of the bilateral kidney of rats in each group at 2 and 4 wk after injection, respectively. Green lines and corresponding quantitative values depict the coronal length diameter of the left renal pelvis. Note the less areas of hydronephrosis was found in the MSCs and MSC‐EVs than the US rat

As listed in Figure 3, the MSCs group and MSC‐EVs treated group showed significantly less hydronephrosis in comparison with the US group at 2 weeks and 4 weeks after treatment. As expected, the Sham group had the lowest values of RPV, whereas the US group had the highest values of these parameters (Table 1). Notably, the RPV values were statistically alleviated in the MSCs as well as MSC‐EVs treatment groups compared to the US group (*P *<* *.01 for all). Furthermore, the MSC‐EVs group had the better alleviation of hydronephrosis when compared to MSCs groups at 2 weeks and 4 weeks, but the difference was not statistically significant (*P *=* *1.000, *P *=* *1.000).

**Table 1:** Comparison of changes of renal pelvic volume among groups at 2 and 4 wk after injection ($\overset{¯}{x}$ ± s)

Group	n	2 wk				4 wk			
		MAD (mm)	MLD (mm)	MTD (mm)	Renal pelvic volume (mm^3^)	MAD (mm)	MLD (mm)	MTD (mm)	Renal pelvic volume (mm^3^)
Sham	8	3.05 ± 1.03	6.68 ± 1.49	3.78 ± 0.84	37.98 ± 12.94	3.05 ± 1.09	8.88 ± 2.74	3.82 ± 1.58	49.63 ± 21.86
US	8	5.81 ± 1.4	15.25 ± 1.24	8.96 ± 1.66	408.38 ± 109.62&	7.49 ± 1.23	25.32 ± 3.41	13.18 ± 1.52	1330.47 ± 427.77&
US + MSCs	7	4.12 ± 0.79	13.56 ± 1.09	5.99 ± 1.25	172.51 ± 39.16& ^,^ §	6.56 ± 1.63	14.6 ± 2.47	9.11 ± 1.58	454.0 ± 145.7# ^,^ §
US + MSC‐EVs	8	3.86 ± 0.99	12.17 ± 2.56	6.16 ± 1.31	145.44 ± 36.78# ^,^ §	6.61 ± 1.58	13.25 ± 2.33	9.05 ± 1.6	414.45 ± 139.98# ^,^ §

US, Ureteral stricture, MSCs, Mesenchymal stem cells, MSC‐EVs, MSCs‐derived extracellular vesicles; MAD, Maximum anteroposterior diameter; MLD, Maximum length diameter; MTD, Maximum transverse diameter; mm, millimeter; Renal pelvic volume, MAD*MLD*MTD*0.523.

^#^ *P *<* *.05 vs the Sham group, ^&^ *P *<* *.01 vs the Sham group, ^§^ *P *<* *.01 vs the US group.

### 3.4 Renal function

As shown in Figure S1A, MSCs and MSC‐EVs rats exhibited lower but not statistically Cr than those in the US group at 4 weeks after injection (*P *=* *1.000, *P *=* *.928). The BUN was significantly lower in both MSCs and MSC‐EVs rats when compared to US group (*P *=* *.000, *P *=* *.000; Figure S1B). No differences in Cr and BUN were observed between MSCs and MSC‐EVs animals (*P *=* *1.000, *P *=* *1.000), indicating that MSC‐EVs treatment had almost the same efficacy on preventing a deterioration of the renal function to that of MSCs.

### 3.5 Histology

**Figure 4:**
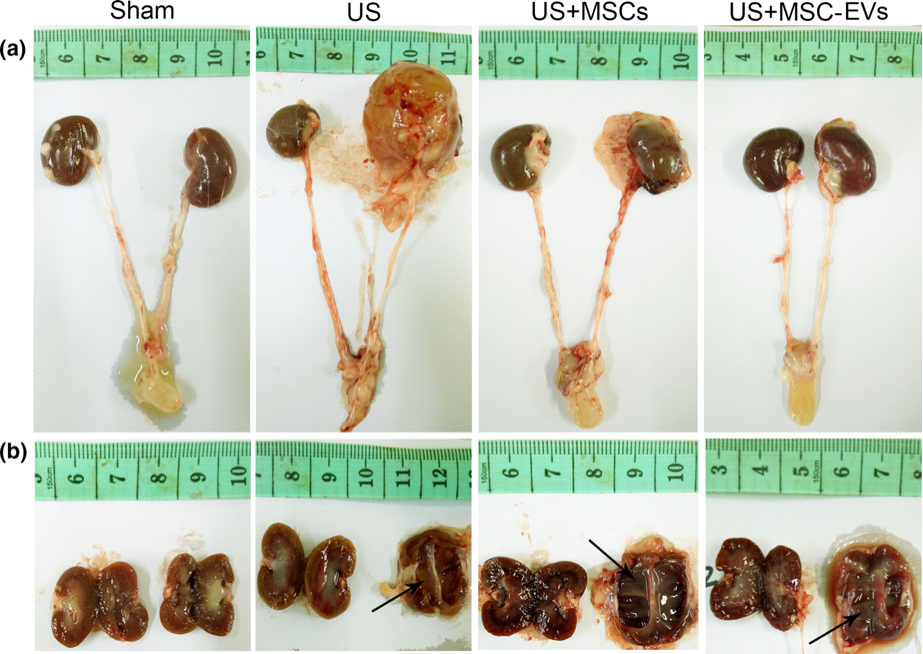
Gross anatomy. Upper panels are representative macrographs of the bilateral kidney, bilateral ureter and bladder of the rats in each group at 4 wk after treatment. Lower panels are longitudinal sections of the bilateral kidney from the same rat. Black arrows indicate sites of pyelectasis and ultrathin renal cortex of the kidney of rats from the US, MSCs and MSC‐EVs group. Note the larger left renal volume and pyelectasis was observed in the US rat

**Figure 5:**
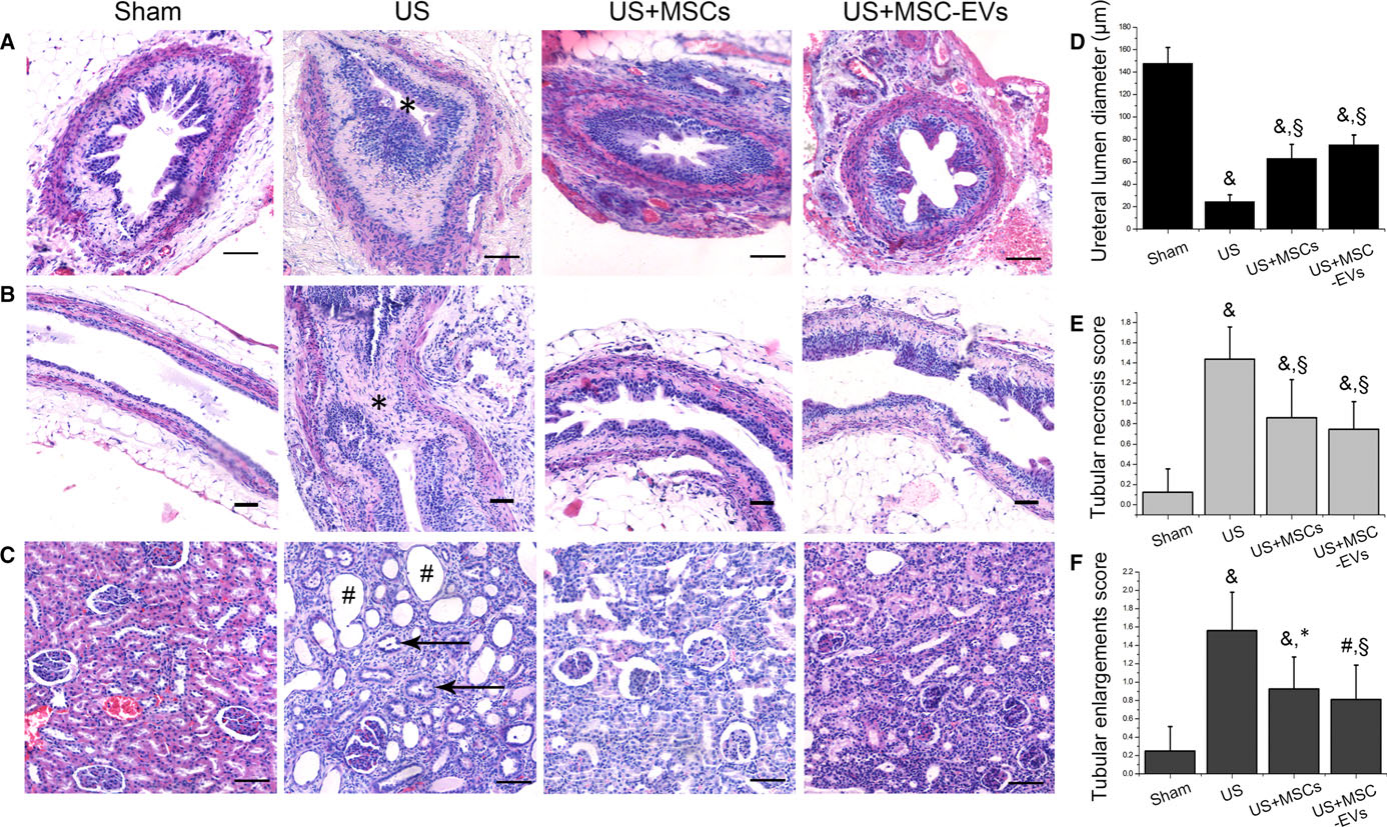
Haematoxylin and eosin (HE) staining. Representative photomicrographs of HE staining in sections of the upper ureter and the homolateral kidney. Scale bar: 50 μm. A, HE staining on cross‐sections (magnification 200×) and B, longitudinal sections of the ureter (magnification 100×) from rats in each group. In the US rats, there is a distinct narrowing of the ureteral lumen (*).C, HE staining on sections of kidney from the same rat in each group (magnification 200×). Note remarkable tubular dilation (#) and glomerulus atrophy (black arrows) was detected in the US rat. D, The quantifications of the ureteral lumen diameter in each group. E, F, Histological scoring of the areas with tubular necrosis and tubular enlargements in each group. ^#^ *P *<* *.05 vs the Sham group, ^&^ *P *<* *.01 vs the Sham group, *P <* *.05 vs the US group. ^§^ *P *<* *.01 vs the US group

Giant unilateral hydronephrosis (Figure 4A) and ultrathin renal cortex (Figure 4B) was observed in the US group, while these morphological changes significantly ameliorated in both MSCs and MSC‐EVs groups. Histological alterations in the cross‐section (Figure 5A) and longitudinal section (Figure 5B) of ureter revealed severe stenosis (narrowing of the ureteral lumen) in US rats, which was significantly alleviated in the MSCs and MSC‐EVs treatment group. The quantifications of the ureteral lumen diameter in each group were summarized in Figure 5D. Changes of renal structure in US rats were characterized by tubular dilation, tubular destroy, apoptosis and glomerulus atrophy, whereas these disorganizations were significantly improved in MSCs and MSC‐EVs treated rats (Figure 5C). Corresponding histological scoring of the areas with tubular necrosis and tubular enlargements in each group were listed in Figure 5E and F.

**Figure 6:**
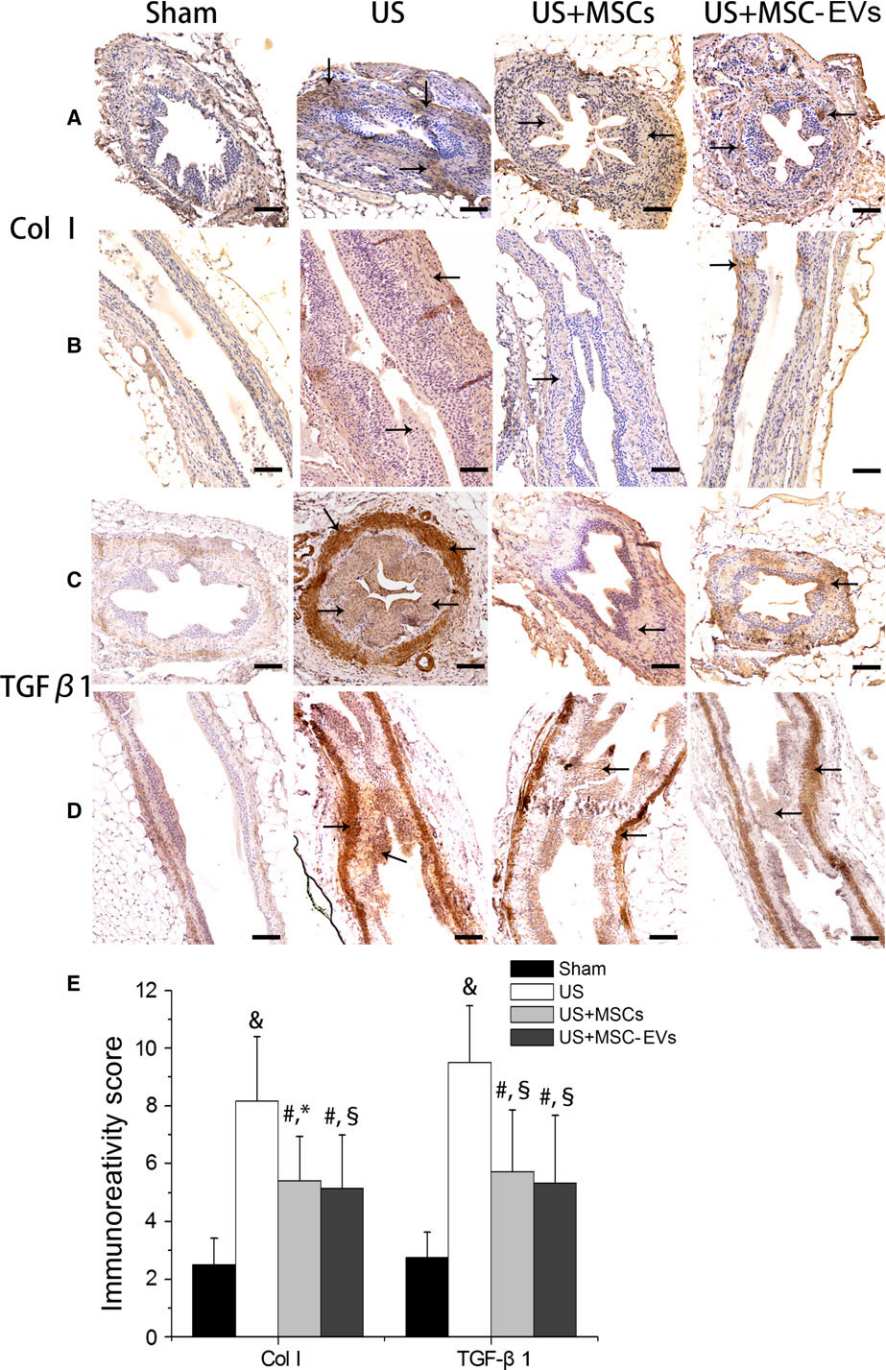
Immunohistochemical analysis. Representative images of immunohistochemical staining for Col I (A & B) and TGF‐β1 (C & D) protein expression in the experimental ureteral sections (A,C cross‐sections, magnification 200×; (B,D) longitudinal sections, magnification 100×) in Sham rat and in US rat with or without MSCs or MSC‐EVs at 4 wk after injection (Scale bar: 50 μm). Arrow indicates positive staining (brown). (E) The immunoreactivity score of Col I and TGF‐β1 in upper ureter tissues in each group, as determined by positive cell percentages and staining intensities. The results were presented as Mean ± standard deviation. ^#^ *P *<* *.05 vs the Sham group, ^&^ *P *<* *.01 vs the Sham group, *P <* *.05 vs the US group, ^§^ *P *<* *.01 vs the US group

TGF‐β1 is a recognized fibrosis factor. Col I, one of the extracellular matrix molecules, is a key biomarker during fibrosis process. The experimental ureter from US rats showed significantly increased levels of Col I and TGF‐β1 expression as compared to the Sham rats (*P *=* *.000, *P *=* *.000); MSCs and MSC‐EVs treated groups showed a significant decrease in the number of Col I and TGF‐β1 positive cells when compared with the US group (*P *<* *.05, for all). However, these immunoreactivity changes were not significantly different between MSCs and MSC‐EVs treatment group (*P *=* *1.000, *P *=* *1.000; Figure 6).

### 3.6 Gene and protein expression

**Figure 7:**
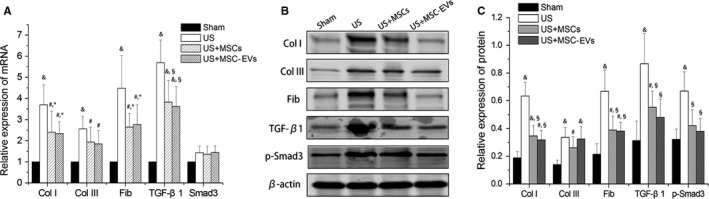
qRT‐PCR and Western blot. Analysis on the upper ureter for fibrosis‐associated gene and protein expression in each group. (A) Relative mRNA expression of Col I, Col III, Fib, TGF‐β1 and Smad3 at 4 wk after injection. Representative Western blots (B) and corresponding densitometry and statistical analysis (C) showing Col I, Col III, Fib, TGF‐β1 and p‐Smad3 protein expression at 4 wk after injection in each group. β‐actin served as the loading control. All results were expressed as means ± standard deviation of the 3 separate experiments. ^#^ *P *<* *.05 vs the Sham group, ^&^ *P *<* *.01 vs the Sham group, *P <* *.05 vs the US group, ^§^ *P *<* *.01 vs the US group. Note both the mRNA and protein levels of Col I, Fib and TGF‐β1 were markedly increased in the US rat, which were reduced by the injection of MSCs or MSC‐EVs

Activation of TGF‐β1/Smad3 pathways is a key mediator not only of epithelial‐mesenchymal transition induction, but also the synthesis of extracellular matrix molecules such as Col I, Fib and Col III, leading to tissue fibrosis. As shown in Figure 7, the US group had significantly higher mRNA and protein levels of Collagen I, Fib, TGF‐β1 and protein level of p‐Smad3 than those of the MSCs and MSC‐EVs treated groups (*P *<* *.05, for all). The differences of Col III expression (both mRNA and protein) and gene expression of Smad3 were not statistically significant among the US, MSCs and MSC‐EVs treated groups (*P *>* *.05, for all). No significant difference in both the mRNA and protein levels of these molecules were found between MSCs and MSC‐EVs rats (*P *>* *.05, for all).

## 4 DISCUSSION

In the present study, we found that transplantation of a bolus dose of MSCs by intra‐arterial route counteracts US formation through the anti‐fibrotic action in a rat model. Importantly, we provided evidence that EVs derived from MSCs might be involved in this function due to similar curative effects of transplantation of MSCs and MSC‐EVs were observed in this study. To our knowledge, this is the first study that investigates the anti‐fibrotic action of MSCs‐derived EVs in US development. MSCs have been therapeutically explored in regenerative medicine for their differentiative and secretory potential. Recently, it was reported that MSCs can modulate gene expression by releasing extracellular microvesicles to orchestrate tissue repair and underpin the maintenance of physiological processes.^22^, ^23^, ^24^ MSC‐EVs might act as the therapeutic vectors regulating multiple fibrogenetic parameters and exerting anti‐inflammatory, antioxidative, restoration of external matrix degradation and proangiogenic properties.^25^, ^26^

Wound healing response including hemostasis, inflammation, proliferation and remodelling that need to restitute the homeostasis thus not to cause an excessive fibrosis and scarring.^27^ Severe or repetitive ureteral injury can evolve into an irreversible fibrotic response which represents excessive accumulation of the epithelium and the surrounding tissues result in a narrowing of the ureteral lumen namely US. Fibrogenesis was characterized by activating and proliferating extracellular matrix‐producing myofibroblasts, which can lead to several chronic pathologies such as permanent scarring and organ malfunction.^28^ Many distinct triggers were identified to contribute to the development of a fibrotic disease. For US, ureteral inflammatory lesions,^29^ ureteric intima ischaemia^30^ and urinary extravasation from the gap of ureter^31^ are considered related to the progression of ureteric fibrosis and malformations. As is known from the clinicopathological feature of severe ureteral injury, the lesion would turn into fibrosis or stricture gradually and cannot be reversed into healthy tissue. Surprisingly, our study demonstrated that administration of MSCs and its derived EVs at an early stage (at the end of the surgery) have the potential therapeutic effect in preventing ureteral fibrosis in a rat model of ureteral injury.

To date, there has not been a standardized method for mimicking and characterizing abnormal wound healing of iatrogenic US formation in an experimental rodent model. Previous models have been used to induce ureteral fibrosis, that is, thermal trauma,^32^ electrical stimulation^33^ and uretero‐ureteral anastomosis.^34^, ^35^ However, none of these techniques could properly generate reproducible stricture formation thereby might incur experimental biases. Recently, Pan et al^6^ proposed a new animal model for ureteral injury and showed that local fixation of hemostat clamp injury on the ureter of rabbits caused distinct ureteral fibrosis. In this study, we employed a withdrawable microscopic vascular clamp placed on the proximal ureter and subsequently removed 6 hours later because it was considered to be a model of ureteral injury characterized by local ischaemia and inflammation and the gradual development of ureteral fibrosis, thereby mimicking the abnormal wound healing of uretero‐ureteral anastomosis or endoureterotomy in humans. We visualized remarkable stricture formation in the model rats with HE and immunohistochemistry staining and consistent ureteral fibrosis was validated by gene and protein expression analyses.

Using this model, we showed that intrarenal‐arterial administration of either a bolus dose of MSCs or MSC‐EVs significantly modified ureteral remodelling and alleviated stricture formation and therefore improved the renal function. As the damage site in the present US model was on the proximal ureter and the upper ureter is supplied by the renal artery, thus we administrated MSCs/MSC‐EVs via the renal arterial route. The anti‐fibrosis effect of MSCs/MSC‐EVs might be associated with inhibition of excessive accumulation of extracellular matrix as shown by the decrease of mRNA and protein expression in Col I and Fib.

TGF‐β1 have been shown to play a critical role in ureteral fibrosis and stricture formation in human and animal studies.^6^, ^36^ TGF‐β1 activation can phosphorylate the downstream receptor‐associated Smads (ie, Smad3), that is the classic TGF‐β1/Smad signalling leading to tissue fibrosis.^37^ In the present study, we found that the stenotic ureter of MSCs and MSC‐EVs groups had a greater than 36% and 45% reduction in the level of TGF‐β1 protein expression compared with the US group as judged by WB. In addition, the protein expression of p‐Smad3 had a trend similar to that of TGF‐β1 protein expression when comparison was made in these groups. These data indicated the reduction of Col I and Fib gene and protein ectopic expression by either MSCs or MSC‐EVs administration might be via inhibiting the TGF‐β1/Smad signalling pathway, thus attenuated ureteral fibrosis. Our finding was consistent with previous studies in which inhibition of TGF‐β1/Smad signalling alleviates urethral fibrosis,^38^ renal fibrosis^39^ and bladder fibrosis.^40^

Herein, we found that intrarenal‐arterial administration of MSCs was sufficient to counteract US formation. Similar effects were observed in MSC‐EVs treatment group, which were accomplished by transplantation of 25 μg EVs preparations corresponding to the amount produced by approximately 3.5 million MSCs. As the dosage of EVs (25 μg) was according to a bolus dose of concentrated MSCs‐conditioned medium corresponding to the amount conditioned by 3.5 million MSCs, we speculated the exact curative effects of MSCs on ureteral injury might attributable to the paracrine factor—MSCs‐derived EVs and not stem cell differentiation into an appropriate cell type and replace damaged or apoptotic cell in the tissue. Interestingly, there are supporting studies that suggested the therapeutic effects of MSCs on tissue regeneration are not facilitated by the direct cellular differentiation and tissue integration, but rather by activating the tissue‐resident recipient cells via paracrine signalling.^41^, ^42^, ^43^

Evidence from the literature suggests that EVs improve the recovery of organic function, enhance angiogenesis and inhibit apoptosis by delivering microRNAs, mRNAs and active proteins to orchestrate tissue repair.^44^, ^45^, ^46^ Also, MSCs‐derived EVs had a potent anti‐inflammatory and pro‐remodelling effect on cell phenotype, acting as the key immunomodulators in biological processes.^15^ The capacity of EVs to halt pro‐fibrotic pathways may have contributed to their specialty for “reprogramming” fibroblasts by residing in their unique microRNA and non‐coding RNAs payload, fundamentally transforming the phenotype of these cells.^47^, ^48^

Analyses of MSCs‐derived EVs nucleic acid content demonstrated shuttle selected pattern of microRNAs may contribute to the mechanism of anti‐fibrotic effect.^49^ Thus, it rises the possibility that MSCs‐derived EVs significantly reduced the ureteral fibrosis of a US model might partly conducted through EVs miRNAs. Therefore, we have made an attempt to explore the implacable miRNA that could be anti‐fibrotic via the GeneCards database (http://www.genecards.org/). Results from the miRTarBase using specific search strategy (keywords: “Bone marrow‐derived mesenchymal stem cells,” “microRNA,” “fibrosis” and “exosome”) showed that the 24 potential miRNAs target TGF‐β1 and might play role in anti‐fibrotic. After reviewing of pertinent literatures, we identified 6 miRNAs (mir‐29b‐3p, mir‐19b‐3p, mir‐130a‐3p, mir‐590‐5p, mir‐146a‐5p and mir‐181a‐5p) were reported have the potency of anti‐fibrotic in the liver, renal, cardiac and pulmonary tissue.^50^, ^51^, ^52^, ^53^, ^54^, ^55^, ^56^, ^57^ Therefore, we envisage the possibility that such potential cargo microRNAs in EVs may involve in the anti‐fibrotic action in US development. As mentioned previously, recurrent US were found after uretero‐ureteral anastomosis and ureteroneocystostomy, our results indicated that these patients (especially in those have suffered repeated surgery before) treated with MSC‐EVs after the surgery immediately might achieve clinical benefit in prevention of strictures.

One limitation of this study was that we used here only one dose and a single dose of either MSCs or MSC‐EVs. Also, injection of MSCs/MSC‐EVs were only performed at an early stage of US development (4 weeks). Last, we failed to track the EVs after transplantation, which could not be fully explained the direct action of EVs in the injury site. Thus, future studies should evaluate dose responses and explore different timings of administration to translate this therapy into clinical research.

## 5 CONCLUSIONS

We successfully established an animal model to mimic experimental rodent model for US. Administration of MSCs might be a promising approach for counteracting US formation. Such therapeutic effect might be due to the release of EVs through paracrine mechanisms of MSCs. The current findings may offer a potential target for clinical therapy of recurrence of strictures after uretero‐ureteral anastomosis or endoureterotomy.

## COMPETING INTERESTS

The authors confirm that there are no conflict of interests.

## AUTHOR CONTRIBUTIONS

Jintai Luo: project development, data analysis and manuscript writing; Shankun Zhao: experiment model establishment, data analysis and manuscript writing; Jiamin Wang: data collection and manuscript writing; Ermao Li and Lianmin Luo: histology examination and data collection; Zhiguo Zhu, Yangzhou Liu and Ran Kang: data analysis; Zhigang Zhao: project development and manuscript editing.
