# Supplementary figures and images for: Bone marrow mesenchymal stem cells reduce ureteral stricture formation in a rat model via the paracrine effect of extracellular vesicles
Source: J Cell Mol Med. 2018 Jul 11;22(9):4449–59. doi: 10.1111/jcmm.13744 (PMC6111875; doi:10.1111/jcmm.13744)

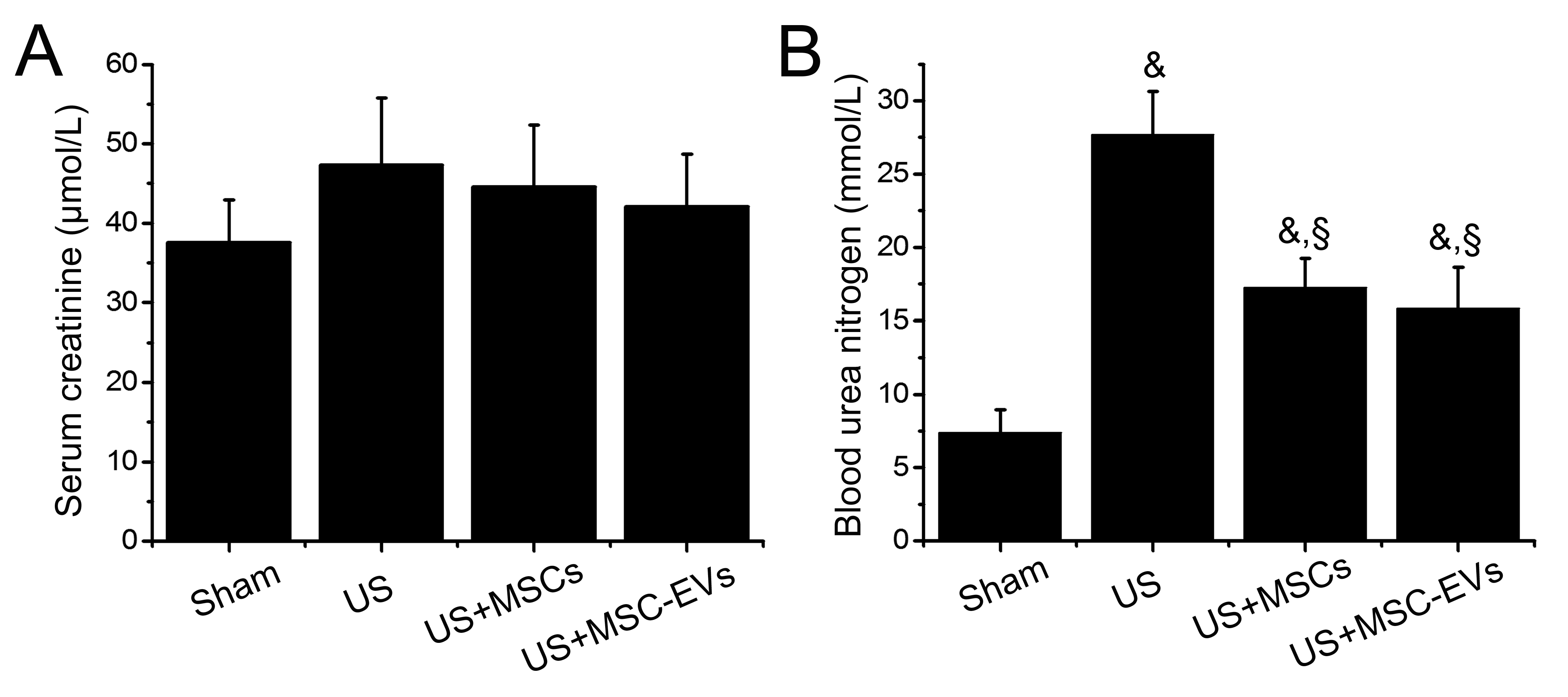

Supplement: Supplementary file 1 [file JCMM-22-4449-s001.tif]
