# Supplementary material for: Bone marrow mesenchymal stem cells reduce ureteral stricture formation in a rat model via the paracrine effect of extracellular vesicles
Source: J Cell Mol Med. 2018 Jul 11;22(9):4449–59. doi: 10.1111/jcmm.13744 (PMC6111875; doi:10.1111/jcmm.13744)
Supplement: Supplementary file 2 [file JCMM-22-4449-s002.doc]

**Supplementary Tab 1.Description of the primer sequences.**

| Primer | Sequence (5’-3’) | Amplicon Size (bp) |
| --- | --- | --- |
| **Col I** |  | 106 |
| Forward | CTCCTGGCAAGAACGGAGA |  |
| Reverse | CCAGCTGTTCCAGGCAATC |  |
| **Col III** |  | 124 |
| Forward | CACAGCCTTCTACACCTGCTCC |  |
| Reverse | CCTGGTTGTCCTGGAAGACC |  |
| **Fib** |  | 266 |
| Forward | CCCCACCTCAGGACTTTTCC |  |
| Reverse | CCGTTGTCAAAACAGCCAGG |  |
| **TGF-β1** |  | 183 |
| Forward | CTACTACGCCAAAGAAGTCACC |  |
| Reverse | GTTGACTTGAATCTCTGCAGGC |  |
| **Smad3** |  | 164 |
| Forward | GACTAGGTGTGAGCCCTTTAC |  |
| Reverse | ATGGTTGACCCACATCCTGGTG |  |
| **β-actin** |  | 93 |
| Forward | GAGAGGGAAATCGTGCGT |  |
| Reverse | GGAGGAAGAGGATGCGG |  |

Col I, collagen I; Col III, collagen III; Fib, fibronectin; TGF-β1, transforming growth factor-β1.
