# Supplementary material for: Bone marrow mesenchymal stem cells reduce ureteral stricture formation in a rat model via the paracrine effect of extracellular vesicles
Source: J Cell Mol Med. 2018 Jul 11;22(9):4449–59. doi: 10.1111/jcmm.13744 (PMC6111875; doi:10.1111/jcmm.13744)
Supplement: Supplementary file 3 [file JCMM-22-4449-s003.doc]

Supplementary Tab 2. Comparison of changes of body weight among groups ($\overline{x}$±s).

| Group | n | Body weight (g) | | |
| --- | --- | --- | --- | --- |
|  |  | initial | 2 weeks | 4 weeks |
| Sham | 8 | 315.38$\pm$11.76 | 334.13$\pm$13.92 | 384.25$\pm$16.32 |
| US | 8 | 312.5$\pm$10.42 | 322.29$\pm$11.49 | 325.88$\pm$13.64^&^ |
| US+MSCs | 7 | 313.0$\pm$9.20 | 322.29$\pm$7.43 | 331.43$\pm$15.68^&^ |
| US + MSC-EVs | 8 | 307.5$\pm$12.34 | 318.63$\pm$10.46 | 336.13$\pm$10.41^&^ |

US = Ureteral stricture, MSCs = Mesenchymal stem cells, MSC-EVs = MSCs-derived extracellular vesicles.

^&^*P*<0.01 vs the sham group.
